# Gamabufotalin triggers c-Myc degradation via induction of WWP2 in multiple myeloma cells

**DOI:** 10.18632/oncotarget.7398

**Published:** 2016-02-15

**Authors:** Zhenlong Yu, Tao Li, Chao Wang, Sa Deng, Baojing Zhang, Xiaokui Huo, Bo Zhang, Xiaobo Wang, Yuping Zhong, Xiaochi Ma

**Affiliations:** ^1^ College of Pharmacy, Academy of Integrative Medicine, Dalian Medical University, Dalian, China; ^2^ Department of Biology, College of Chemistry and Life Sciences, Zhejiang Normal University, Zhejiang, China; ^3^ Department of Neurosurgery, The Second Affiliated Hospital of Dalian Medical University, Dalian, China; ^4^ Department of Hematology, Beijing Chaoyang Hospital, Capital Medical University, Beijing, China

**Keywords:** gamabufotalin, multiple myeloma, c-Myc, degradation, WWP2

## Abstract

Deciding appropriate therapy for multiple myeloma (MM) is challenging because of the occurrence of multiple chromosomal changes and the fatal nature of the disease. In the current study, gamabufotalin (GBT) was isolated from toad venom, and its tumor-specific cytotoxicity was investigated in human MM cells. We found GBT inhibited cell growth and induced apoptosis with the IC50 values <50 nM. Mechanistic studies using functional approaches identified GBT as an inhibitor of c-Myc. Further analysis showed that GBT especially evoked the ubiquitination and degradation of c-Myc protein, thereby globally repressing the expression of c-Myc target genes. GBT treatment inhibited ERK and AKT signals, while stimulating the activation of JNK cascade. An E3 ubiquitin-protein ligase, WWP2, was upregulated following JNK activation and played an important role in c-Myc ubiquitination and degradation through direct protein-protein interaction. The antitumor effect of GBT was validated in a xenograft mouse model and the suppression of MM-induced osteolysis was verified in a SCID-hu model *in vivo*. Taken together, our study identified the potential of GBT as a promising therapeutic agent in the treatment of MM.

## INTRODUCTION

Multiple myeloma (MM) is an aggressive tumor characterized primarily by the accumulation of abnormal plasma cells in the bone marrow. MM is the second most prevalent hematologic neoplasm in the world, accounting for approximately 1% of neoplastic diseases and 13% of hematological malignancies [1–3]. It is considered a progressive and incurable disease with a poor prognosis in the majority of patients. The prognosis for a given individual with MM differs largely depending on pathological phenotype. The overall median survival of MM patients is 3-4 years with conventional chemotherapy [1–3]. Moreover, patients with MM frequently develop severe complications, including infection, anaemia, thrombocytopenia, renal failure, and bone disease. This destructive bone disease, which is primarily attributed to enhanced activation of osteoclasts and the infiltration of MM cells in the bone marrow, reduces mobility and increases risk of pathological fractures and paralysis.

MM is a highly heterogeneous disease with very complex cytogenetic and genetic aberrations. The inevitable accumulation of genetic errors across different cellular pathways drives the malignant change of MM toward a more aggressive phenotype. Basic studies have characterized potential pathways and targets crucial for the disease maintenance and progression [4–6]. Treatment strategies targeting IL-6 or other autocrine cytokines and growth factors have been effective in preclinical and clinical trials of MM. Other candidate targets, including cyclin D1, c-Myc, mTOR, CD40 and Hsp90, have been extensively investigated in the pathogenetic and preclinical studies. It has been well known that c-Myc pathway is activated in more than 60% of the patient-derived MM cells [7–9]. Targeting c-Myc by shRNA or small molecule inhibitors were shown to be promising to MM therapy [7, 8]. Despite advances in pathological mechanism and treatment, MM largely remains incurable with high mortality rate. The average 10-year overall survival rate currently is only approximately 17% for all ages [1–3]. Therefore, development of novel therapeutic approaches would be beneficial for MM treatment and would improve patient outcomes.

Natural products are an important treasure-trove to provide leading compounds for developing novel agents with superior activity and less toxicity. The Chinese traditional medicine *ChanSu* has been considered a prospective candidate due to emerging evidence for its effectiveness in tumor treatment [10]. *ChanSu*, also known as toad venom, is a dried product of the skin and parotid venom glands from the Asiatic toad (*Bufo gargarizans*). However, *ChanSu* is a complex mix of chemicals with poor drugablitity including clinically toxicity and insolubility in water. It is important to identify the active components of *ChanSu* and their targets in tumor cells for the development of optimized analogues. So far, a group of more than 100 bufadienolides, including cinobufagin, bufalin, bufotalin, gamabufotalin (GBT) and resibufogenin, are separated and identified to be the major active components with antitumor activities in *ChanSu* [10]. Several mechanisms of action for bufadienolides to antagonize tumor progression have been proposed, including the inhibition of heat shock protein 27 (HSP27), Topo II, and Survivin; the induction of Tiam1 and p21; mitochondrial calcium overload; and upregulation of proapoptotic Bax and Fas [11–16].

GBT is a newly identified natural product and derived from *ChanSu* in our lab. There is only limited information available on its growth inhibitory effects in solid tumors *in vitro* and the mechanisms have been largely unexplored. In the current study we isolated and characterized the bioactive GBT, and studied its growth inhibitory effects against MM *via* targeting c-Myc regulatory network.

## RESULTS

### GBT suppresses cell viability and triggers apoptosis in MM cells

GBT was successfully isolated and identified in our lab (Supplementary Figure 1). And GBT exhibited superior metabolic stability and excellent safety profile (Supplementary Figure 2).

**Figure 1:**
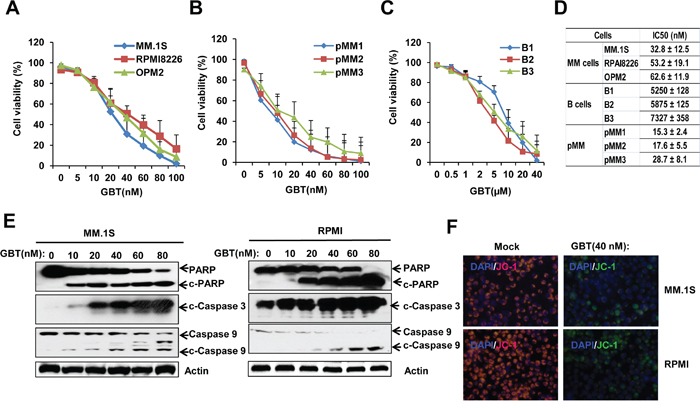
GBT reduced cell viability and induces apoptosis in MM cells Cell viability was reduced with increasing concentrations of GBT in **A.** 3 MM cell lines; **B.** 3 primary CD138^+^ cells; **C.** 3 B cells from healthy donors; **D.** The IC_50_ values of GBT in myeloma and B cells; **E.** GBT induces cleavage of caspases and PARP in MM.1S and RPMI 8226 cells, indicative of apoptosis; **F.** JC-1 staining assay showing green fluorescent apoptotic MM.1S and RPMI 8226 cells. Data represent mean ± SEM from three independent experiments.

In order to evaluate the anti-myeloma effects of GBT, cell viability was tested in MM cell lines, CD138^+^ cells separated from MM patients, and in normal B-cells. After GBT treatment, all the three MM cells (MM.1S, RPMI 8226, and OPM2), showed dose-dependent decrease in the cell viability (Figure 1A). In addition, primary CD138^+^ cells from three different MM patients also showed decreased viability in a dose-dependent manner (Figure 1B). The nanomolar concentrations of GBT caused a dose-dependent decrease in the viability of MM cell, while did not induce any significant changes in the normal B-cell viability (Figure 1C). IC_50_ values of primary MM cells, MM cell lines, and B-cells are indicated in Figure 1D. Accordingly, the IC_50_ of GBT was around 50 nM in MM cells, 20 nM in primary MM cells, and >5000 nM in the normal B cells. These data indicate that GBT-mediated cytotoxicity is tumor-specific and excludes normal cells.

Further, GBT induced-apoptosis in MM cells was also confirmed. As expected, GBT triggered the cleavage of caspase-3/9 and PARP in MM.1S and RPMI 8226 cells (Figure 1E). Measurement of mitochondrial membrane potential (MMP) using JC-1 staining illustrated that GBT treatment resulted in mitochondrial damage and MMP loss in MM.1S and RPMI 8226 cells (Figure 1F). Our results indicate that GBT causes mitochondria-dependent apoptosis selectively in the malignant MM cells, and excludes cytotoxic effects on the normal cells.

### GBT suppresses cell-cycle regulatory proteins and induces cell-cycle arrest

**Figure 2:**
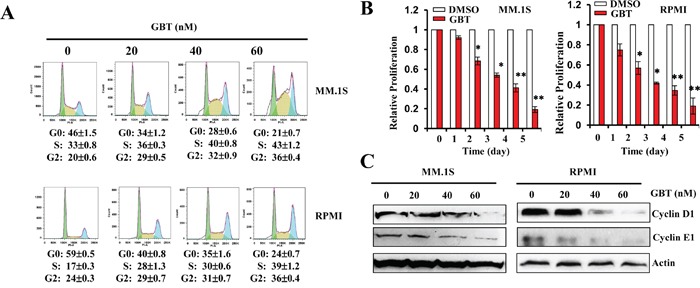
GBT inhibits MM cell proliferation and induces cell-cycle arrest **A.** GBT induces S and G2/M cell-cycle arrest in MM.1S and RPMI 8226 cells exposed to different concentrations of GBT for 24 h; **B.** Cell proliferation was measured by MTS assay after GBT (50 nM) treatment for the indicated time periods; **C.** Changes in cell-cycle regulatory proteins after GBT treatment for 24 h. Data represent mean ± SEM from three independent experiments. Statistical significances at **p<0.005 *vs.* respective vehicle.

Our results indicated that besides apoptosis, GBT significantly induced cell-cycle arrest in the MM cells. As shown in Figure 2A, a substantial proportion of GBT-treated cells were growth-arrested at the S checkpoint in a dose-dependent manner. Meanwhile, GBT treatment also caused the accumulation of MM cells in G2/M phase. The anti-proliferative effect of GBT was also confirmed using MTS assay, where cell proliferation of MM.1S and RPMI 8226 cells were decreased by 80% at 50 nM (Figure 2B). These data provide strong evidence that GBT inhibits proliferation of MM cells by inducing S and G2/M phase cell-cycle arrest.

Next, to ascertain detailed mechanisms underlying GBT-induced cell-cycle arrest, several proteins involved in S and G2/M arrest were evaluated by western blotting. GBT treatment greatly decreased protein levels of cyclin D1 and cyclin E1, the crucial rate-limiting factors governing S and G2/M progression during cell multiplication (Figure 2C). The loss of cyclin D1 and cyclin E1 expression might attribute to the cell cycle arrest.

### GBT specifically targets c-Myc network

**Figure 3:**
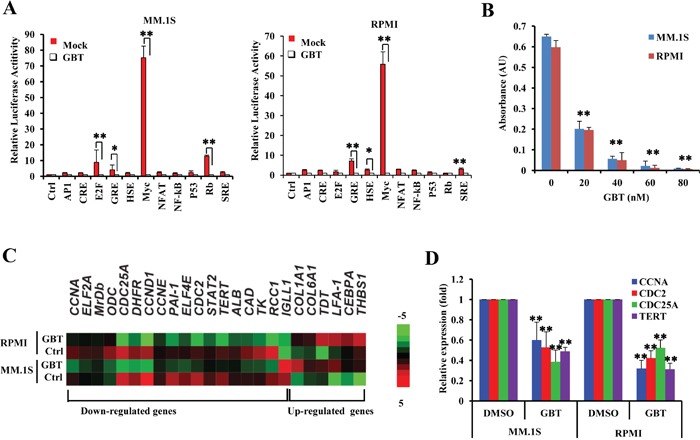
GBT targets c-Myc-mediated transcription **A.** Luciferase activity assay of 11 transcriptional factors in the presence of GBT; **B.** ELISA-based DNA-binding assays to determine the effect of GBT (50 nM) on selective depletion of nuclear c-Myc; **C.** Heatmap of clustered gene expression data from multiplex experiments (Nanostring) of cancer-associated genes in two human MM cell lines treated with GBT or vehicle control; **D.** Real-time qPCR showing the expression of genes downstream of c-Myc in MM.1S and RPMI 8226 cells treated with 50 nM of GBT. Data represent mean ± SEM from three independent experiments. Statistical significances at **p<0.005 and *p<0.05 *vs.* respective vehicle/mock.

To further identify the pharmacological targets of GBT, we applied a screening experiment, using the luciferase reporter system to monitor the activities of 11 different transcriptional factors involved in growth regulation or stress responses. The results showed that GBT mildly interrupted E2F, GRE, MYC, and Rb activities in MM.1S or GRE, HSE, MYC and SRE activities in RPMI 8226 cells (Figure 3A). Notably, c-Myc activity was greatly suppressed by more than 70-fold in both MM cells after 50 nM of GBT treatment. In parallel, a gradual decline of chromatin bound c-Myc with increasing concentrations of GBT was observed by nuclear ELISA transcription factor-binding assays (Figure 3B).

We also evaluated the transcriptional consequences of GBT treatment in MM.1S and RPMI 8226 cells utilizing gene expression microarray. Intriguingly, the expression of *c-MYC* was almost unaffected by GBT in this results. Among the GBT-repressed genes, we found that 18 gene sets were annotated as either known or as predicted positive targets of c-Myc, such as *CCNA1*, *CCND1*, *CCNE1*, *CDC2*, *CDC25A*, *EIF2A*, *EIF4E*, *DHFR*, and *TERT*. Six of the c-Myc negative target genes including *CEBPA* were increased after GBT treatment (Figure 3C). Real-time PCR further validated at least four of the decreased genes (Figure 3D). These results strongly suggest that GBT treatment renders a selective abrogation of transcriptional networks mediated by c-Myc, which in part contributes to the growth inhibition mediated by GBT in MM.

### GBT hyperubiquitinates the c-Myc oncoprotein

**Figure 4:**
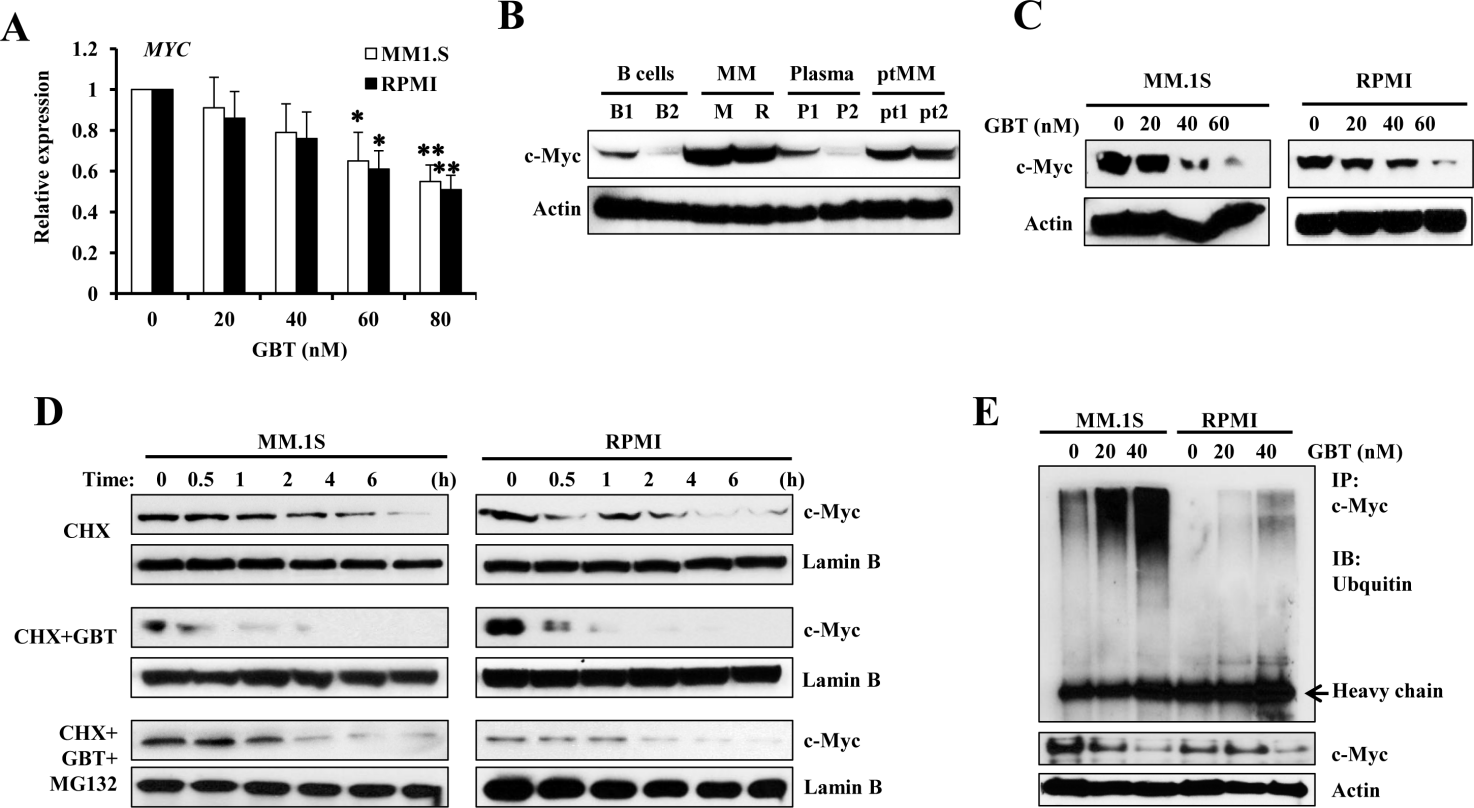
GBT promotes c-Myc degradation **A.** qPCR analysis for *c-MYC* expression in MM cell lines; **B.** Western blotting analysis showing the dose-dependent effect of GBT on c-Myc protein levels in MM cells; B cells, plasma, and MM cells from patients; **C.** Accelerated degradation of c-Myc protein after GBT treatment in MM.1S and RPMI8226 cells; **D.** MM.1S and RPMI8226 cells were treated with 50 nM of GBT, 20 μM of CHX, or the combination of GBT+CHX with 0.5 μM of MG132 for 6 h; **E.** Immunoprecipitation assay showing enhanced ubiquitination of c-Myc in MM.1S and RPMI8226 cells after GBT treatment. Aliquots from the above groups were directly analyzed by Western blot to test for c-Myc protein (bottom panel) in each group. Data represent mean ± SEM from three independent experiments. Statistical significances at **p<0.005 and *p<0.05 *vs.* respective vehicle/mock.

Although GBT targeted the c-Myc network, only high dosage of GBT directly reduced the c-Myc mRNA levels, because only concentrations over 80 nM could induce significant decreases in MYC mRNA levels in MM.1S and RPMI8226 cells (Figure 4A). Indeed, c-Myc protein levels were highly expressed in the two MM cell lines and two patient samples compared with the B cell or plasma control, respectively (Figure 4B). In contrast, GBT treatment effectively decreased the c-Myc protein levels (Figure 4C). It seemed that GBT directly interrupts the stability of c-Myc rather than represses its expression. To verify this, MM.1S and RPMI 8226 cells were treated with 50 nM of GBT, in the presence of a protein synthesis inhibitor, cycloheximide (CHX), and cell extracts were isolated at specific time points and subjected to immunoblotting for the detection of c-Myc degradation. The half-life of c-Myc in MM.1S and RPMI 8226 cells in the presence of CHX alone was about 2 hours (Figure 4D, upper panel), while it was shortened to within 0.5 h on addition of GBT (Figure 4D, middle panel). Further, MG-132, an inhibitor of ubiquitin-mediated protein degradation significantly reversed the effect on c-Myc degradation induced by GBT (Figure 4D, lower panel). Thus, our results showed that GBT significantly accelerated the degradation of c-Myc protein. Meanwhile, another portion of these MM cells were immunoprecipitated by anti-c-Myc antibody and immunoblotted for the ubiquitination assay. Data suggested that GBT promoted c-Myc ubiquitination in a dose-dependent manner (Figure 4E, upper panel), while total c-Myc was decreased with increasing concentration of GBT (Figure 4E, lower panel).

### WWP2 due to GBT stimulation promotes c-Myc polyubiquitination via direct interaction

**Figure 5:**
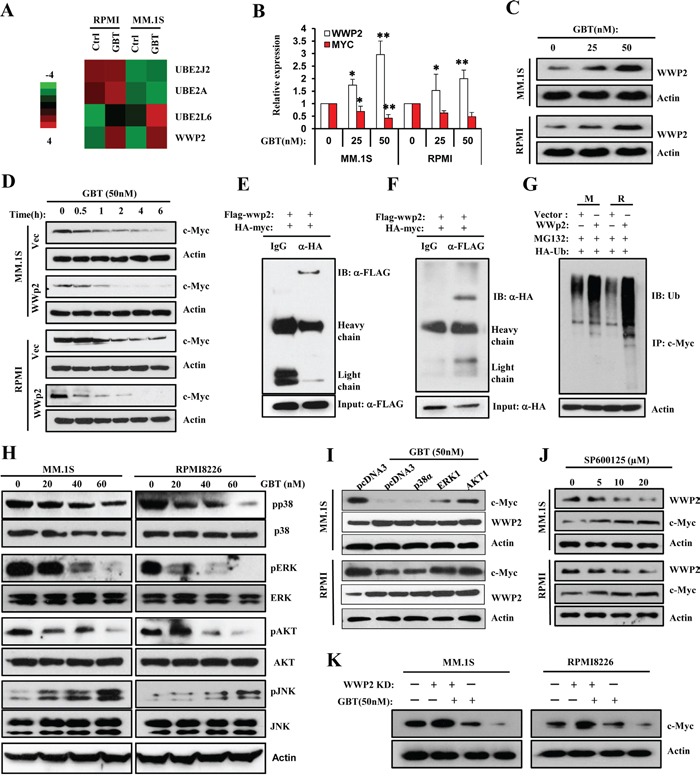
GBT upregulates WWP2 to accelerate c-Myc ubiquitination via JNK cascade **A.** Heatmap of selected genes participating in protein processing and proteolysis in two human MM cell lines treated with GBT or vehicle control; **B.** and **C.** Real-time qPCR and Western blotting analysis confirm the upregulation of WWP2 in MM.1S and RPMI8226 cells treated with increasing concentrations of GBT; **D.** Degradation rates of endogenous c-Myc substrates in MM.1S and RPMI8226 cells transfected with pcDNA3-flag-*WWP2* in the absence and presence of GBT (50 nM). **E.** and **F.** Interaction between WWP2 and c-Myc *in vivo*. 293T cells were transfected with expression vectors for HA-c-Myc and FLAG-WWP2. Cell lysates were subjected to immunoprecipitation with anti-FLAG or anti-HA, and the resulting precipitates were subjected to immunoblot analysis with anti-HA or anti-FLAG, respectively. A portion of cell lysates corresponding to 5% of the input for immunoprecipitation was also subjected directly to immunoblot analysis. Normal IgG was used as a negative control. **G.** Promotion of c-Myc ubiquitylation by WWP2. MM.1S and RPMI8226 cells were transfected with expression vectors or FLAG-WWP2 together with HA-tagged ubiquitin. Immunoprecipitation was performed with anti-c-Myc and subsequently immunoblotting analysis was conducted with anti-HA antibodies to detect c-Myc ubiquitylation. **H.** Effects of GBT on multiple signaling pathways in MM.1S and RPMI8226 cells. Levels of p-AKT/AKT, p-p38/p38, p-ERK/ERK, and p-JNK/JNK in total cell lysates were evaluated by Western blot analysis. β-actin was used as the loading control; **I.** The rate of degradation of endogenous c-Myc on treatment with GBT in MM.1S cells transfected with p38α, ERK1, or AKT1 plasmids. **J.** Inhibition of JNK cascade by increasing concentrations of SP600125 antagonized the upregulation of WWP2 and reversed c-Myc degradation. All data are representative of at least three independent experiments. **K.** c-Myc levels in MM.1S and RPMI8226 cells infected with WWP2-shRNA lentivirus for 48 hr and then treated with or with GBT (50 nM).

GBT responsiveness also uncovered significant alterations (fold>2) of genes associated with protein processing and proteolysis, such as *UBE2J2*, *UBE2L6*, *UBE2A*, and *WWP2* (Figure 5A). Among them, *WWP2,* an E3 ubiquitin-protein ligase, was the most significantly increased gene (>3 fold, *p*<0.001). Real-time PCR (Figure 5B) and immunoblotting (Figure 5C) further confirmed that GBT induced an increase in WWP2 expression. WWP2 functions as a known HECT-domain-containing E3 ligase, controlling ubiquitin-dependent degradation of extensive substrates [21]. Subsequently, we examined whether the upregulated WWP2 participated in c-Myc ubiquitination. Indeed, overexpression of pcDNA3-FLAG-WWP2 in MM.1S and RPMI8226 cells resulted in significantly increased endogenous c-Myc protein degradation, compared with the vector control respectively (Figure 5D).

To characterize whether c-Myc is a direct target of WWP2, we tested the interaction between these two proteins by co-immunoprecipitation assay. As shown in Figure 5E, exogenously expressed HA-tagged c-Myc interacted specifically with FLAG-tagged WWP2 in HEK293T cells; reciprocal coimmunoprecipitation assays also validated the above finding (Figure 5F). Thus, WWP2 might be a physiologically relevant E3 ligase for c-Myc. We further assessed the significance of the c-Myc-WWP2 interaction in MM cells using ubiquitylation assays. MM.1S and RPMI8226 cells were transiently cotransfected with either pcDNA3-flag-WWP2 or vector along with HA-tagged ubiquitin plasmids. As expected, c-Myc was predominantly polyubiquitylated by WWP2 (Figure 5G). Therefore, these results support the rationale that c-Myc is a downstream substrate of WWP2 and GBT-induced upregulation of WWP2 mediates c-Myc ubiquitylation and degradation. Indeed, aberrant overexpression of *c-MYC* is a usual feature of MM; examples were seen in analysis of gene expression pattern from 320 patients in Broyl's database [22] and 247 patients in Dichens's database [23] (Supplementary Figure 3). Interestingly, both Broyl's and Dickens's research revealed a commonly decreased expression of *WWP2* mRNA in primary MM cells (Supplementary Figure 4). Furthermore, in order to verify the significance of WWP2 on GBT induced c-Myc degradation, we next disturbed WWP2 expression using shRNA lentivirus then the cells were treated with or without GBT. As shown in Figure 5K, knockdown of WWP2 considerably altered c-Myc protein level in GBT-treated cells, compared to the non-target control cells. These data implied that WWP2 was a target of GBT to trigger c-Myc degradation.

### GBT activates JNK cascade to upregulate WWP2

Since the stabilization and degradation of c-Myc is controlled by ordered phosphorylation of multiple cascades including MAPK and AKT, we examined the activation status of MAPK and AKT kinases. As shown in Figure 5H, decreased phosphorylation of AKT and ERK kinases was evident at 20 nM of GBT treatment, while higher concentration moderately suppressed the phosphorylation of p38 kinase. In comparison, JNK cascade was greatly activated by 20 nM of GBT treatment. Overexpression of AKT1 and ERK1 plasmids partially ameliorated the degradation of c-Myc protein, but failed to block WWP2 upregulation (Figure 5I). Nevertheless, JNK inhibition by SP600125 effectively repressed WWP2 expression and rescued the protein level of c-Myc (Figure 5J). These results indicate that the activation of JNK cascade is a determinant factor for the induction of WWP2, thereby affecting c-Myc ubiquitylation and degradation.

### GBT inhibits the growth of MM xenograft in SCID mice

**Figure 6:**
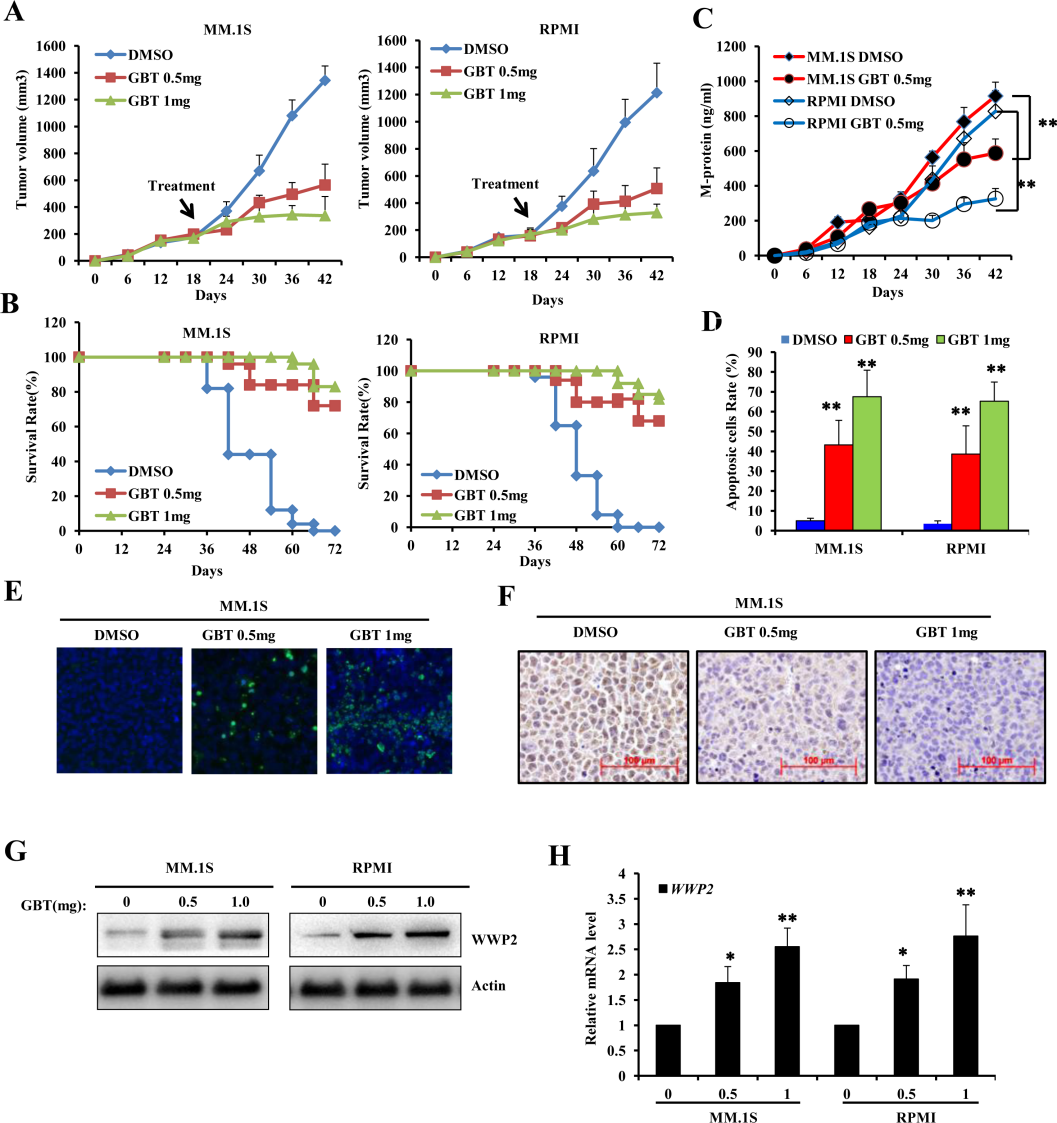
GBT suppresses MM xenograft tumors in mice model **A.** Tumor volume showing trends of xenograft growth in MM.1S and RPMI8226 cells in a SCID mice model; **B.** Survival rates following GBT treatment in MM xenograft models; **C.** ELISA assay to detect M-protein levels in the sera of xenografts developed from MM.1S or RPMI8226 cells; **D.** Quantitative analysis of the apoptotic MM cells from xenograft tumors treated with different dose of GBT; **E.** TUNEL assay of the xenograft tumor tissues; **F.** Representative immunohistochemical staining of c-Myc in different xenograft tissues. **G.** WWP2 protein level and **H.** *WWP2* mRNA level were examined in the xenograft tissues. Scale bars = 100 μm. Data represent mean ± SEM (n=12/group). Statistical significances at **p<0.005 *vs.* vehicle-treated group.

*In vivo* anti-tumor activity of GBT was measured in immunodeficient SCID mice. The observed rates of tumor growth were significantly decreased in GBT groups compared with control group (*P*<0.05; Figure 6A). Similarly, the average size of MM cell-derived xenograft tumors in mice treated with GBT was significantly lower than that of control group. As a result of delayed tumor progression, GBT improved survival outcome without any adverse reactions in the mice (Figure 6B). Consistently, the level of circulating human M-protein secreted by malignant MM cells, which often represents the MM tumor burden, was found to be lower in the GBT treatment group compared with control group (Figure 6C). In addition, quantitative analysis of apoptosis by *in situ* TUNEL staining revealed that more MM cells succumbed to apoptosis on increasing doses of GBT (Figure 6D). The percentage of apoptotic cells in both MM groups receiving GBT was greater than 42%, and reached a peak level of about 74% on treatment with 1 mg/kg of GBT. Representative TUNEL images from MM.1S tumor tissues treatmented with GBT were shown in Figure 6E. As expected, GBT greatly decreased the expression of c-Myc and its target genes, revealing a major mechanism of suppression of MM xenografts as demonstrated by IHC (Figure 6F) and real time PCR (Supplementary Figure 5), respectively. Simultaneously, the WWP2 protein levels and mRNA levels were found increased in the MM-derived xenograft tissues (Figure 6G, 6H). Therefore, our current *in vivo* data demonstrates robust anti-tumor effects of GBT in mice challenged with MM.

### GBT attenuates myeloma-induced bone destruction *in vivo*

**Figure 7:**
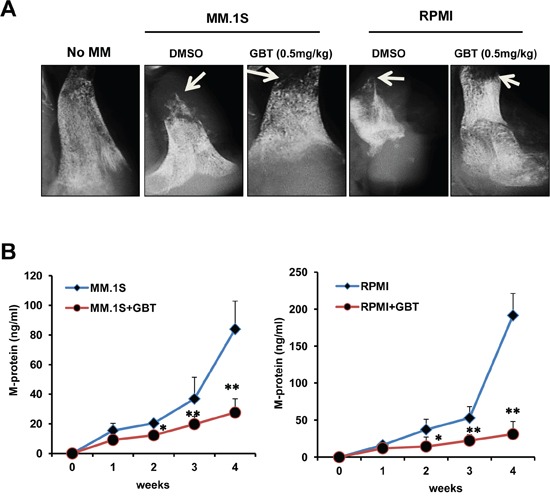
GBT alleviates bone destruction induced by MM cells in a SCID-hu mice model **A.** X-ray of the bone resorption induced by MM.1S or RPMI8226 cells in the DMSO control group or GBT group (0.5 mg/kg) in a SCID-hu mice model; **B.** ELISA assay of the M-protein levels from sera of SCID-hu mice with MM.1S or RPMI8226 cells in the DMSO control group or GBT group (0.5 mg/kg). Data represent mean ± SEM (n=12/group). Statistical significances at **p<0.005 and *p<0.05 *vs.* respective vehicle-treated group.

Bone destruction is a hallmark of MM in most patients as MM stimulates production of activated osteoclasts [24]. Herein, we investigated whether GBT ameliorates myeloma-induced bone destruction in the SCID-hu mice model. Our results demonstrated that osteolytic bone lesions developed in all of the myeloma bearing mice irrespective of GBT treatment. Compared with vehicle control, mice treated with GBT had significantly fewer lytic bone lesions (Figure 7A). In parallel, the amount of circulating M-protein was also lower in the GBT-treated mice (Figure 7B). Thus, these results strongly suggest the potential of GBT not only in the treatment of MM, but also in the prevention of myeloma-induced lytic bone destruction.

## DISCUSSION

Bufadienolide derivatives such as GBT, bufalin (BF), cinobufagin (CB), and resibufogenin (RB), from Chinese medicine *Chansu*, have provided therapeutic benefits to a wide range of tumors involving human astrocytoma, leukemia, prostate, liver, lung and gastric tumors [11–16]. Among them, GBT showed better metabolic stability and safety in our previous experiments. In previous studies, we have found that GBT suppressed cell growth in several human lung cancer cell lines with IC_50_ less than 100 nM, while had no obvious cytotoxicity on human normal cell lines [25, 26]. In the current study, we found that GBT exhibited strong anti-proliferative effects on various MM cells in a dose- and time-dependent manner. The IC50 values for the established MM cell lines and primary CD138+ MM cells were about 30-60 nM, while it was more than 5000 nM in the CD19+/CD138- B cells, indicating that GBT is selectively potent in malignant MM cells rather than in the normal cells, thereby limiting the side effects of GBT treatment. Therefore, GBT could be regarded as a highly specific therapeutic agent with robust potential against MM.

Oncogenes of the MYC family, including c-Myc, N-Myc and L-Myc, are master regulators of cell proliferation, growth, survival and differentiation [27, 28], because of its transcriptional repression function [29]. MYC is one of the most highly amplified and frequently translocated oncogene among a variety of human cancers, such as, multiple myeloma [30, 31]. The expression of the N-Myc and L-Myc genes is confined to particular stages of embryonic development, and in the adult to immature cells of the hematopoietic and neuronal compartments [32, 33]. In contrast, the *c*-Myc gene is expressed during all stages of the cell cycle in dividing cells. And c-Myc mediates various physiological functions including cell cycle control, apoptosis, protein synthesis, and cell adhesion [27, 34]. Overexpression of c-Myc is associated with the features of many human malignancies, such as proliferation, invasion, angiogenesis, and metastasis [34, 35]. Thus, in our study, when Myc gene was down-regulated by GBT treatment, we believed that it was c-Myc, actually. And our data also showed that GBT treatment inhibited c-Myc activity, that in part contributing to the growth inhibition mediated by GBT in MM.

More importantly, our results from the luciferase reporter experiments showed that c-Myc network is a crucial target affected by GBT. Oncogene *MYC*, encoding a transcriptional factor recognizing a specific subset of E-box elements (CACGTG), plays a central role in multiple tumors by regulating cell proliferation, survival, and chemoresistance. Previous studies have implicated that MM patients with aberrant activation of c-Myc tend to exhibit inferior clinical outcomes. Using microarray analysis, we identified several GBT-responsive genes. 5,952 genes were downregulated after GBT treatment, whereas 2,482 genes were upregulated by more than 2-fold. Among them, *CCNA1*, *CCND1*, *CCNE1*, *CDC2*, *CDC25A*, *EIF2A*, *EIF4E*, *DHFR*, and *TERT* have been previously reported as major c-Myc targets [36–39]. Otherwise, C/EBPα, a particularly potent regulator of cell cycle exit, was upregulated after GBT exposure as it is specifically downregulated by c-Myc [40]. Combined with the validation of real-time PCR experiments, and gene expression profiles revealed that GBT treatment impaired the c-Myc-mediated gene program, contributing to cell cycle arrest and apoptosis.

The striking benefit of inhibiting c-Myc functions has stimulated significant research efforts in drug discovery [34, 41, 42]. Using specific shRNA, antisense oligonucleotide or small molecule inhibitors strategies that can specifically prevent the recruitment of various transcription factors and hence lead to the downregulation of c-Myc transcription, was shown to be lethal in a number of tumor cell lines [43–45]. Herein, we found that GBT can induce c-Myc ubiquitination and subsequent degradation. Previous studies have established that phosphorylation at Ser62 and Thr58 was observed with constitutive c-Myc stabilization by ERK kinase and GSK3β [44–47]. In this study, the concerted inhibition of both the ERK and AKT cascades provides a feasible explanation for GBT-induced c-Myc ubiquitination and subsequent degradation. We also found that JNK activation in GBT treatment was another determinant factor for c-Myc degradation since the JNK cascade induced the expression of WWP2.

WWP2, a known HECT-domain-containing E3 ubiquitin ligase, regulates ubiquitin-dependent degradation of its substrates. It has been reported that WWP2-mediated ubiquitylation is involved in the regulation of multiple substrates, such as Notch3, PTEN, OCT4 and TRIF [48–51]. Our results showed that c-Myc is a downstream substrate of WWP2, and wild-type WWP2 not the catalytically inactive mutant facilitates c-Myc polyubiquitylation. Indeed, c-Myc stability is governed by a complicated network, but not all of E3 ubiquitin ligases are equivalent in their capacity to control c-Myc ubiquitination and degradation [52, 53]. In the presented study, our results indicate that JNK-induced WWP2 is an E3 ubiquitin ligase responsible for c-Myc degradation under stress conditions. Our results suggest that WWP2 is a negative regulator of c-Myc. Thus, manipulating WWP2 is a promising approach to antagonize c-Myc.

Meanwhile, the potent antitumor activity of GBT against MM xenografts was further confirmed in SCID mice. Remarkably, mice tolerated the therapy of GBT without weight loss or any other life threatening toxicities. It appeared that the high specificity of GBT for c-Myc was responsible for the effectiveness and the lesser toxicity. Besides, GBT also has additive therapeutic benefits as evidenced by the decreased bone destruction. Our results showed that GBT could directly attenuate osteoclast differentiation *via* blocking the expression of multiple cytokines, which impacts tumor outgrowth and osteoclast differentiation [54, 55]. As a consequence of the inhibition of both tumor progression and osteoclastogenesis, GBT treatment alleviated bone destruction in myeloma-implanted SCID-hu mice.

Taken together, our results highlight that GBT could serve as a lead compound for its development as an inhibitor for c-Myc pathway and is selectively useful in c-Myc-dependent tumors. GBT can exert negative effects on c-Myc stability and function via upregulating WWP2. Our study sheds light on the manipulation of c-Myc ubiquitination and degradation to be a viable strategy to counteract tumor progression.

## MATERIALS AND METHODS

### Detection of mitochondrial membrane potential (DCm)

Loss of mitochondrial membrane potential of MM cells was measured using the MitoProbe JC-1 assay (Molecular Probes, Invitrogen). Briefly, MM cells were treated with the GBT (50 nM) for 24 hours and then incubated with JC-1 (5μM) at 37°C for 30 minutes in the darkness. After being washed with PBS for three times, cells were analyzed immediately using a Zeiss 4.4.0 Axiovert 200 Inverted Fluorescence Microscope with a 100 W mercury lamp following conditions: 330-385 nm excitation filter (excf), 400-nm dichroic mirror (dm), and 420-nm barrier filter (bf), for Hoechst 33258; 450-480 nm excf, 500-nm dm, and 515 nm bf for JC-1.

### Luciferase assay and qPCR

MM cells were transfected with 0.9 μg of Pathway Profiling Luciferase plasmids (Mountain View, CA) and 0.1 μg of pRL-TK Renila plasmid (Promega, Madison, WI) for 48h, and luciferase activity was measured using Dual-Luciferase Reporter assay system (Promega, WI) or Pathway Profiling SEAP System (Clontech, Mountain View, CA) according to the manufacturers' protocols. For qPCR 1 μg of total RNA was subjected to reverse transcription in a 25 μL system, and 1 μL of the final cDNA was applied to real-time PCR amplification using the primers listed as following: *c-Myc*: F-CTACCCTCTCAACGACAGCA, R-TCTTGTTCCTCCTCAGAGTCG; *CCNA*: F-CTCC TGGTGAACAAGCTCAA; R-TGAACTTCACATCTG TGGCA; *CDC-2*: F-TTTCAGAGCTTTGGGCACT, R-AGAGCAAATCCAAGCCATTT; *CD25A*: F-AGTG AGACTTCCTGCCTCGT, R-GGCCACTGCTACCTGG TACT; *TERT*: F-CTCCATCAGAGCCAGTCTCA; R-TT CACCTGCAAATCCAGAAA; *WWP2:* F-CGGACCA CCTCACCTACTT; R-TCTTATTGAGCATCCGCTTG; *GAPDH*: F-CTGGGCTACACTGAGCACC, R-AAG TGGTCGTTGAGGGCAATG.

### Immunoprecipitation and ubiquitination assay

Myeloma cells were appropriately treated with GBT (50nM) and lysed with RIPA buffer. Clarified lysates were precleared with Protein G-Sepharose (Pierce, Rockford, IL), and then immuno-precipitated with anti-HA, anti-flag, or antic-Myc absorbed to protein G-Sepharose. Proteins were fractionated by SDS-polyacrylamide gel electrophoresis and transferred to PVDF membranes (Millipore, MA) and blotted with anti-flag, anti-HA, or anti-Ubiquitin antibodies, respectively. 293T cells were transfected with different plasmids encoding FLAG-WWP2, His-c-Myc, or HA-ubiquitin.

### DNA-binding enzyme-linked immunosorbent assay

DNA binding was quantified with a specific ELISA format method as modified for c-Myc-specific detection according to the manufacturer's instructions (KeyGEN Biotech, China). A BioTrak II plate reader spectrophotometer (Amersham Biosciences/GE Healthcare, Piscataway, NJ) was used to obtain absorbance readings (A450 nm).

### Gene Expression Microarray and Exon Array Data Analysis

Affymetrix Exon array data were processed using the GeneBASE software [17]. Affymetrix microarray data were processed using the RMA algorithm to compute gene expression levels [18]. Agilent data were normalized. To determine differentially expressed genes, gene expression levels were log2 transformed and then analyzed using limma [19].

### Animal studies, Immunohistochemistry and TUNEL assay

CB.17 SCID mice were used, and the studies were approved by the Institutional Animal Care and Use Committee at Dalian Medical University. SCID-hu mice model was established according to previous report [20]. Eight mice per group were subcutaneously inoculated with 5×10^6^ MM cells. Tumor volume (mm^3^) was measured every 3 days in two dimensions using a caliper and was calculated as 0.4 × (short length)^2^ × long length. Sera were collected every 6 days from the mice during treatment and tested for MM-secreted M-proteins or their light chains using ELISA. Mice were humanely euthanized when they became moribund or when the subcutaneous tumors reached 15 mm in diameter. All specimens were subject to immunohistochemistry (IHC) analysis using the Enovision Detection Kit/DAB (DAKO A/S, Denmark) according to the manufacturer's protocol with anti-c-Myc antibody. Apoptosis was measured using the ApopNexin Biotin Apoptosis Detection Kit (Intergen, Purchase, NY). For TUNEL assay, sections were deparaffinized, rehydrated, and retrieved. DNA strand breaks were labeled with dUTP to the 3′-OH sites and catalyzed by TdT at 37°C. Samples treated with DNase I were used as positive controls. Sections were counterstained with hematoxylin.

Cell culture, cell proliferation, cell cycle and apoptosis assay, and western blotting are provided in *SI Materials and Methods.*

### Statistical analysis

All data are shown as means ± standard deviations. Student's t test was used to compare various experimental groups; significance was set at P value less than 0.05.

## SUPPLEMENTARY DATA FIGURES


