# Supplementary material for: Gamabufotalin triggers c-Myc degradation via induction of WWP2 in multiple myeloma cells
Source: Oncotarget. 2016 Feb 15;7(13):15725–37. doi: 10.18632/oncotarget.7398 (PMC4941272; doi:10.18632/oncotarget.7398)
Supplement: Supplementary file 1 [file oncotarget-07-15725-s001.pdf]

SUPPLEMENTARY DATA

SI MATERIALS AND METHODS

Gamabufotalin isolation and identification

Gamabufotalin was isolated from *ChanSu*, which was secreted from the postauricular and skin glands of *Bufobufogargarizans* Cantor, by Dr. Ma Xiaochi Lab (Dalian Medical University, Liaoning, China). The crude materials of *ChanSu* were purchased from Qingdao (Shandong, China), and a voucher specimen had been deposited at School of Pharmacy, Dalian Medical University. A preparative high-speed counter-current chromatography (HSCCC) method for isolation and purification of bufadienolides from *ChanSu* was developed by using a stepwise elution with two-phase solvent system composed *n*-hexane-ethyl acetate-methanol-water at the ratios of 4:6:2:4 (v/v), 4:6:2.5:4 (v/v) and 4:6:3.2:4 (v/v). A total of 68 mg of GBT was obtained in one-step separation from 1.5 g of the crude extract with purity of 99%. Its chemical structure was identified on the basis of ^{1}H-NMR, ^{13}C-NMR and ESIMS technology.

Primary myeloma cells and myeloma cell lines

CD138^{+} myeloma cells were isolated by magnetic bead sorting from patient bone marrow. Patients were signed standard consent forms before any procedures were initiated. The study was approved by the Institutional Review Board at Dalian Medical University. All myeloma cell lines were purchased from the ATCC (Rockville, MD). Myeloma cell lines MM.1S, OPM2, and RPMI8266 were purchased from the American Type Culture Collection (Rockville, MD). All myeloma cells were cultured in RPMI-1640 medium supplemented with 10% FBS (Invitrogen, Carlsbad, CA).

Cell proliferation, cell cycle and apoptosis assay

MM cells proliferation, cell cycle and apoptosis analysis were performed as previously described.[1] Briefly, for cell proliferation assay, MM cells were incubated with GBT for different times (1-5 days), then incubated for 1 hour with a cell proliferation assay kit solution (Promega), and finally measured at a 490 nm wavelength; for apoptosis assay, MM cells were incubated with GBT for 48 h, after treatments, myeloma cells were washed in 1×PBS and incubated at room temperature for 20 minutes with the FITC-conjugated Annexin V antibody (Invitrogen, CA, USA) and propidium iodide (Sigma-Aldrich, MO, USA). After incubation and washing, samples were analyzed on a LSR Fortessa (Becton Dickinson), and data were analyzed with FlowJo software;

Lentivirus infection

Lentivirus was packaged in the HEK293T cells. Briefly, the target gene plasmids and the package vectors were co-transfected into the HEK cells using Lipofectamine 2000 reagent (Life Technology, CA, USA), 6 hours later the culture media were changed for collection every 24 hours. All supernatant was concentrated to 1:10 by volume for infection of MM cells. The WWP2 shRNA plasmid for lentivirus package was purchased from Santa Cruz (Santa Cruz Biotechnology, Dallas, TX, SUA).

Western blotting

50 μg of total protein was loaded. Antibodies against PARP, caspase-3, caspase-9, c-Myc, ubiquitin, p-p38, p-AKT, p-JNK, and p-ERK were purchased from Cell Signaling Technology. The Cyclin D1, Cyclin E, WWP2, and Actin antibodies were purchased from Santa Cruz. Anti-Flag and anti-His tag antibodies were from Sigma Aldrich.

SI REFERENCES

1. Liu Z, Li T, Jiang K, Huang Q, Chen Y, Qian F. Induction of chemoresistance by all-trans retinoic acid via a noncanonical signaling in multiple myeloma cells. *PLoS One*. 2014;9:e85571.

SUPPLEMENTARY FIGURES

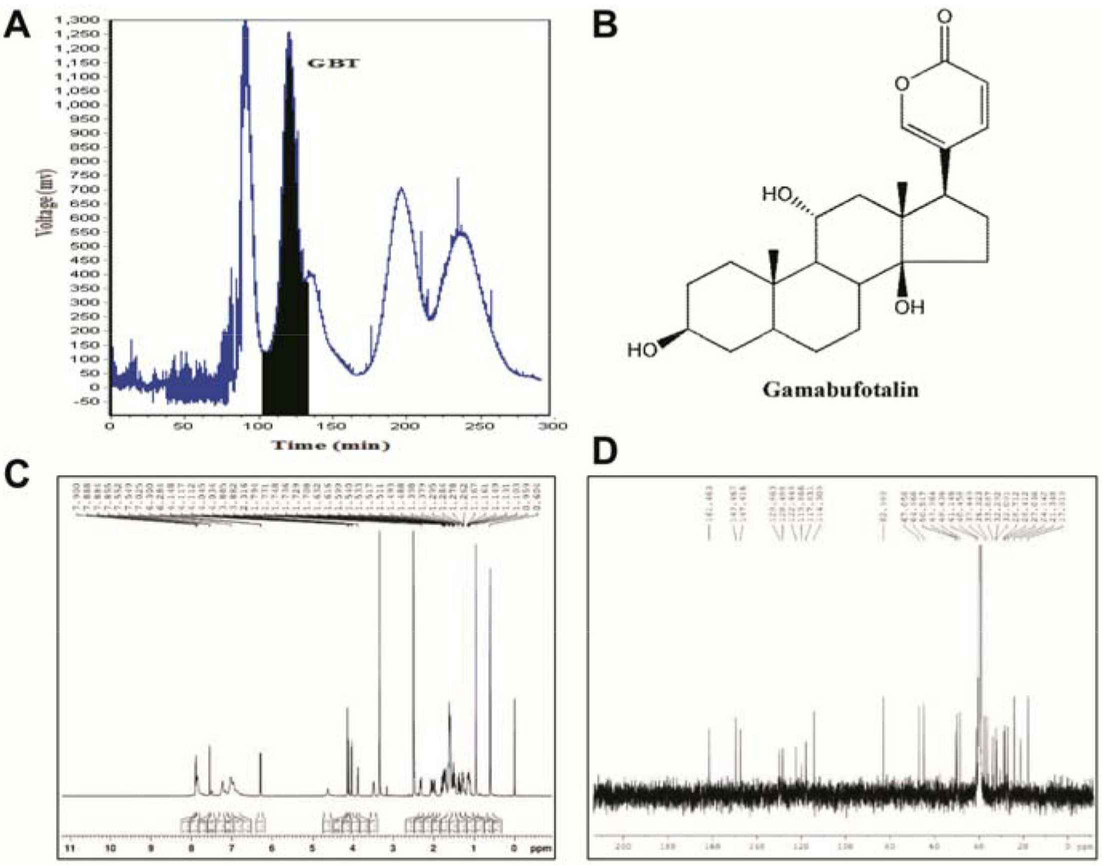

Supplementary Figure S1: Chemical characterization of GBT. A. HSCC chromatogram showing the peak of GBT obtained from the crude extract of *ChanSu*; **B.** Chemical structure of GBT; **C.** ^{1}H-NMR spectra of GBT; **D.** ^{13}C-NMR spectra of GBT.

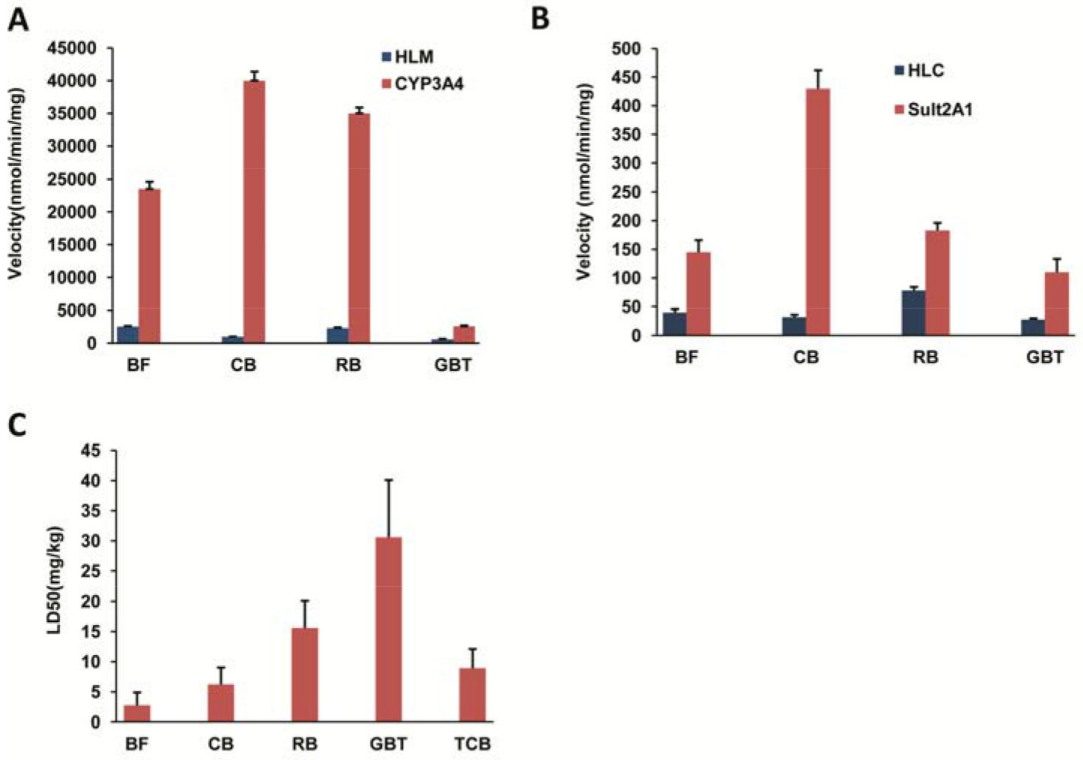

Supplementary Figure S2: The metabolic stability and safety of several major bufadienolides from *ChanSu*. **A.** 5 β-hydroxylation metabolism rates of CB, BF, RB, and GBT by HLMs and CYP3A4 (Phase I metabolism); **B.** 3-sulfation of CB, BF, RB, and GBT by HLC and SULT2A1 (Phase II metabolism); **C.** LD_{50} values of CB, BF, RB, GBT and TCB in mouse (mg/kg, i.p.). CB: Cinobufagin; BF: Bufalin; RB: Resibufogenin; GBT: Gamabufotalin; TCB: Telocinobufagin. Data represent mean ± SEM from three independent experiments.

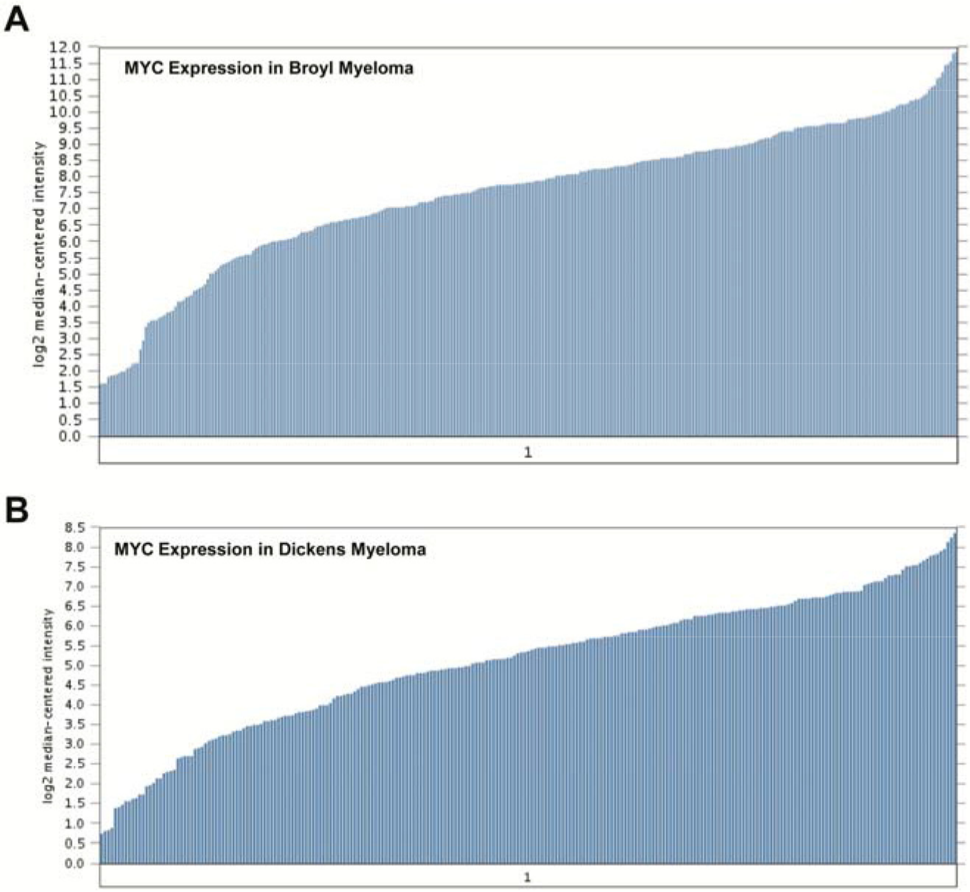

Supplementary Figure S3: *MYC* gene expression in patients with MM. *MYC* expression levels in **A.** 320 patients in Broyl's database and **B.** 247 patients in Dichens's database array analysis using human genome array.

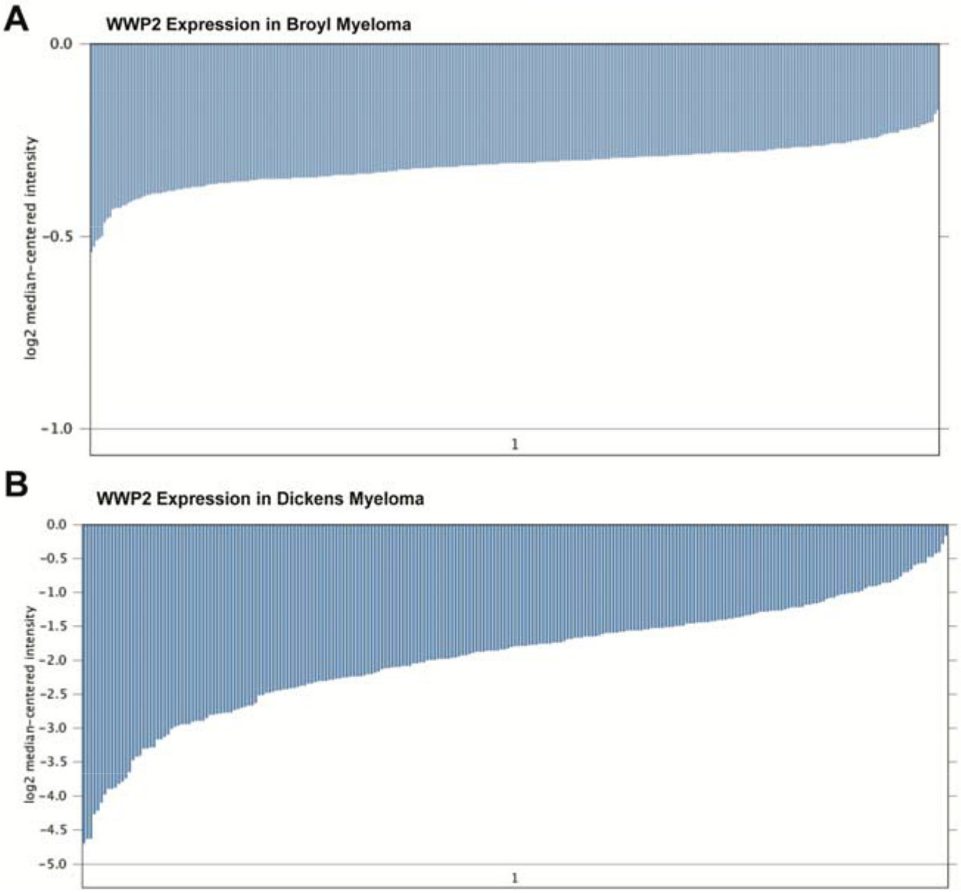

Supplementary Figure S4: WWP2 gene expression in patients with MM. *WWP2* expression levels in **A.** 320 patients in Broyl's database and **B.** 247 patients in Dichens's database array analysis using human genome array.

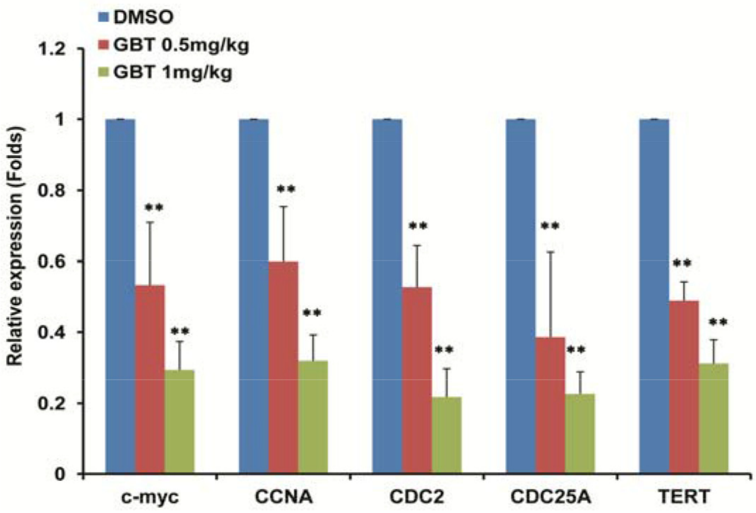

Supplementary Figure S5: Expression of *MYC* and the other target genes in the MM xenograft tumors treated with different doses of GBT. Data represent mean ± SEM (n=12/group). Statistical significances at $^{**}p<0.005$ *vs.* vehicle-treated group.
